# Cassini observations of ionospheric plasma in Saturn's magnetotail lobes

**DOI:** 10.1002/2015JA021648

**Published:** 2016-01-25

**Authors:** M. Felici, C. S. Arridge, A. J. Coates, S. V. Badman, M. K. Dougherty, C. M. Jackman, W. S. Kurth, H. Melin, D. G. Mitchell, D. B. Reisenfeld, N. Sergis

**Affiliations:** ^1^ Mullard Space Science LaboratoryUniversity College LondonDorkingUK; ^2^ Centre for Planetary Sciences at UCL/BirkbeckLondonUK; ^3^ Department of PhysicsLancaster UniversityLancasterUK; ^4^ Space and Atmospheric Physics Group, The Blackett LaboratoryImperial College LondonLondonUK; ^5^ Department of Physics and AstronomyUniversity of SouthamptonSouthamptonUK; ^6^ Department of Physics and AstronomyUniversity of IowaIowa CityIowaUSA; ^7^ Department of Physics and AstronomyUniversity of LeicesterLeicesterUK; ^8^ The Johns Hopkins University Applied Physics LaboratoryLaurelMarylandUSA; ^9^ Department of Physics and AstronomyUniversity of MontanaMissoulaMontanaUSA; ^10^ Office for Space ResearchAcademy of AthensAthensGreece

**Keywords:** Saturn, Cassini, ionospheric outflow, magnetosphere, lobe, magnetotail

## Abstract

Studies of Saturn's magnetosphere with the Cassini mission have established the importance of Enceladus as the dominant mass source for Saturn's magnetosphere. It is well known that the ionosphere is an important mass source at Earth during periods of intense geomagnetic activity, but lesser attention has been dedicated to study the ionospheric mass source at Saturn. In this paper we describe a case study of data from Saturn's magnetotail, when Cassini was located at ≃ 2200 h Saturn local time at 36 *R*_*S*_ from Saturn. During several entries into the magnetotail lobe, tailward flowing cold electrons and a cold ion beam were observed directly adjacent to the plasma sheet and extending deeper into the lobe. The electrons and ions appear to be dispersed, dropping to lower energies with time. The composition of both the plasma sheet and lobe ions show very low fluxes (sometimes zero within measurement error) of water group ions. The magnetic field has a swept‐forward configuration which is atypical for this region, and the total magnetic field strength is larger than expected at this distance from the planet. Ultraviolet auroral observations show a dawn brightening, and upstream heliospheric models suggest that the magnetosphere is being compressed by a region of high solar wind ram pressure. We interpret this event as the observation of ionospheric outflow in Saturn's magnetotail. We estimate a number flux between (2.95 ± 0.43) × 10^9^ and (1.43 ± 0.21) × 10^10^ cm^−2^ s^−1^, 1 or about 2 orders of magnitude larger than suggested by steady state MHD models, with a mass source between 1.4 ×10^2^ and 1.1 ×10^3^ kg/s. After considering several configurations for the active atmospheric regions, we consider as most probable the main auroral oval, with associated mass source between 49.7 ±13.4 and 239.8 ±64.8 kg/s for an average auroral oval, and 10 ±4 and 49 ±23 kg/s for the specific auroral oval morphology found during this event. It is not clear how much of this mass is trapped within the magnetosphere and how much is lost to the solar wind.

## Key Points

- Tailward observation of ionospheric plasma in the magnetotail lobe
- Evidence for simultaneous reconnection in the plasma sheet
- First observational estimate at Saturn of mass source rate from the ionosphere to the magnetotail

## 1 Introduction

Saturn's magnetosphere is a complex multicomponent plasma system with several internal plasma sources in addition to the solar wind. The largest internal plasma source is from photoionization and electron impact ionization of neutral water and nitrogen molecules from the icy moon Enceladus. These ions are subsequently processed by photolytic and radiolytic processes to produce H^+^ and a variety of water group ions such as OH^+^ and O^+^ that are collectively referred to as W^+^. The other natural satellites, the rings, and Saturn's atmosphere are minor internal sources. The solar wind also plays a role as an external plasma source. A number of studies have focused on the moons, rings, and solar wind as plasma sources, to constrain the extent to which they drive the system. In this paper we provide the first in situ constraints on the role that the ionosphere plays as a mass source for Saturn's magnetotail, via the first observation of ionospheric outflow at a giant planet.

### 1.1 Plasma Sources and Transport in Saturn's Magnetosphere

*Shemansky et al.* [1993] presented Hubble Space Telescope (HST) observations of an OH torus extending from 3 to 8 *R*_*S*_ (1 *R*_*S*_=60,268 km). They identified Enceladus, and to a lesser extent the other icy moons, as H_2_O sources for the magnetosphere. *Jurac et al.* [2002] and *Richardson and Jurac* [2004] estimated the amount of H_2_O needed to maintain the OH cloud and found that a source rate of 3.75 × 10^27^ H_2_O molecules/s (112 kg/s) was required to maintain this cloud, of which 93 kg/s must be coming from the orbit of Enceladus. This estimate sits within a range of estimated rates between 10^26^ and 10^28^ molecules/s (≃35–350 kg/s) [*Tokar et al.*, 2006; *Waite et al.*, 2006; *Hansen et al.*, 2006]. The large variability in these figures may be a natural result of the time variability of the Enceladus source. Following processing of these neutrals by neutral‐plasma chemistry, the total plasma source rate is around 60–100 kg/s [*Fleshman et al.*, 2013].

Titan has also been studied as a source of mass for Saturn's magnetosphere. *Johnson et al.* [2009] estimates a total ion loss rate from Titan of 1–5 ×10^26^ amu/s (0.16–0.83 kg/s). *Coates et al.* [2012] estimated a loss rates of (8.9, 1.6, 4.0) ×10^25^ amu/s for three crossings of Titan's tail, for an average loss rate of 0.8 kg/s.

Saturn's main rings have an O^+^ and O ${}_{2}^{+}$ atmosphere which can be ionized and act as a mass source for the magnetosphere [*Tokar et al.*, 2005; *Johnson et al.*, 2006a, 2006b; *Bouhram et al.*, 2006; *Luhmann et al.*, 2005; *Martens et al.*, 2008; *Tseng et al.*, 2010]. The ring atmosphere was predicted to vary seasonally as the incidence angle of the solar radiation on the main rings varies seasonally [*Tseng et al.*, 2010]. Using a photochemical model and Cassini plasma spectrometer (CAPS) data, *Elrod et al.* [2012] demonstrated that observed changes in the ring plasma over time were due to seasonal change in the production of neutrals from Saturn's ring atmosphere. We are not aware of any published estimates of the mass loading rate due to the rings.

Plasma produced in the inner magnetosphere from these sources is transported to the outer magnetosphere. This transport is regulated by the centrifugally driven interchange instability [*Mauk et al.*, 2009, and references therein]. The most detectable signature of this process is the injection of hot plasma into the inner magnetosphere accompanied by magnetic pressure enhancements or deficits [e.g., *Hill et al.*, 2005; *André et al.*, 2005, 2007; *Thomsen*, 2013].

The solar wind and ionosphere are thought to be secondary sources, but the source rates have only been estimated, and there are no observational constraints. To estimate the magnitude of the solar wind source, a common approach is to multiply the solar wind mass flux *n*_SW_ *v*_SW_ by the cross‐sectional area of the magnetosphere to obtain an upper limit for the source rate: $n_{\text{SW}}v_{\text{SW}}\pi R_{0}^{2}$. An efficiency factor *O*(10^−3^) is included to account for diversion of the solar wind and magnetosheath plasma around the magnetosphere and the ability of magnetosheath plasma adjacent to the magnetopause to enter the magnetosphere [*Hill*, 1979; *Hill et al.*, 1983; *Vasyliuñas*, 2008; *Bagenal and Delamere*, 2011]. Applying this logic with a solar wind number density between 0.002 and 0.4 cm^3^, and a solar wind speed between 400 and 600 km/s [*Crary et al.*, 2005] with a magnetopause of cross‐sectional area *π*(30*R*_*S*_)^2^ [using the terminator radius of the magnetopause from *Kanani et al.*, 2010], gives an upper limit of between 8.21 × 10^27^ and 2.46 × 10^30^ protons/s (hence between about 13 and 4119 kg/s). Combined with the efficiency factor of 10^−3^, the solar wind is a minor source.

### 1.2 Ionospheric Outflow From Saturn's Atmosphere

The physical mechanisms which lead to the ionosphere outflowing into space were theorized before the ionospheric outflow was detected at Earth. *Dessler and Michel* [1966] and *Bauer* [1962] argued that since the magnetospheric tail has a lower pressure than the ionosphere, there should be a continuous escape of thermal plasma from the ionosphere into the tail (referred to just H^+^ and He^+^ at Earth). By analogy with the solar wind, *Axford* [1968] suggested that this flow should be supersonic and named it the polar wind.

The classical polar wind is an ambipolar outflow of thermal plasma from the high‐latitude ionosphere. The faster upflowing electrons create a charge separation with the more gravitationally bound ions, generating an ambipolar electric field that accelerates the ions to achieve charge neutrality. The plasma, traveling and then escaping the topside of the ionosphere, undergoes four transitions: from chemical to diffusion dominance, from being subsonic to supersonic, from a collision dominated to a collisionless regime, a transition from heavy to light ions (at Earth O^+^ and H^+^) since the light ions are less gravitationally bound.

A steady state polar wind outflow is highly improbable. Magnetospheric electric fields make the ionosphere‐polar wind system convect constantly across the polar region, polar cap, nightside auroral oval, nighttime trough, and sunlit hemisphere. When the magnetic activity increases, plasma convection speeds and particle precipitations intensify. Three‐dimensional time‐dependent simulations of the global ionosphere and polar wind have shown that when the geomagnetic activity changes, the temporal variations and horizontal plasma convection affect the polar wind and its dynamics. Three‐dimensional models (a global ionosphere‐polar wind model) studied how much a geomagnetic storm (for different solar cycles conditions) would have influenced the atmospheric system [*Ganguli*, 1996; *Schunk and Nagy*, 2009, and references therein]. Polar wind outflow increases with geomagnetic activity.

Different mathematical approaches have been used over the years to model the complexity of the polar wind, such as hydrodynamical and hydromagnetic modeling, generalized transport, and kinetic models. Also, numerous studies have been conducted of the nonclassical polar wind, which may contain, for example, ion beams or hot electrons. A wealth of processes might be acting in the polar wind, and still understanding is needed [*Ganguli*, 1996].

Observational evidence of the polar wind at Earth was presented by *Hoffman* [1970] using data from Explorer 31 showing field‐aligned H^+^ with speed ≃ 10 km/s, and flux ≃10^8^ cm^−2^ s^−1^ above 2500 km altitude. Using ISIS 2 and OGO data, similar results for H^+^ were obtained, plus O^+^ and He^+^ observations were added to the picture by *Brinton et al.* [1971], *Taylor and Walsh* [1972], *Hoffman et al.*[1974], *Taylor and Cordier* [1974], and *Hoffman and Dodson* [1980]. More recently, *Chandler et al.*[1991] measured ion density, velocity, and flux variations of polar wind outflows using DE 1 data.

Electron temperature anisotropies, the relationship between the plasma pressure gradient between the ionosphere and deep magnetosphere, and the process of ambipolar diffusion along magnetic field lines was established using Akebono data [*Abe et al.*, 1993a, 1993b; *Yau et al.*, 1995]. Observations of ionospheric outflow in the magnetosphere are harder to make given the temperature of the plasma and charging of the spacecraft. Using Cluster data, *Engwall et al.* [2009a, 2009b] inferred a total outflow from Earth's polar ionosphere of the order of 10^26^ ions/s, which confirmed previous simulation results arguing for the continuous presence of a low‐energy ion population in the lobes. In addition, they inferred that the solar wind dynamic pressure and interplanetary magnetic field played a role in influencing these populations in the lobes.

The polar wind is an important source of plasma in Earth's magnetosphere during periods of geomagnetic activity. The extent of the ionosphere as a plasma source at Saturn has been investigated using numerical models [*Frey*, 1997; *Glocer et al.*, 2007]. These models solve the field‐aligned gyrotropic transport equation [*Gombosi and Nagy*, 1989] for ions and electrons and simulate multiple convecting field line solutions.

*Glocer et al.* [2007] applied this model to Saturn, adapting the chemistry for the composition of Saturn's thermosphere. The model considers the behavior of H^+^ and H ${}_{3}^{+}$. It assumes a stationary neutral atmosphere and models a range in altitude from 1400 (chemical and thermal equilibrium) to 61,000 (lower pressure) km. The background neutral atmosphere required as an input relies on analysis of the stellar occultation measurements (low latitude) of the Voyager 2 Saturn flyby, presented by *Smith et al.* [1983]. The estimates for temperature and density of the neutrals were made at low latitudes; therefore, the model counts for the uncertainty on these parameters with a wide array of temperatures (420–1500 K) which takes into account the possible density and temperature variations from low to high latitudes. From this model, *Glocer et al.* [2007] estimate the polar wind number flux of 7.3 × 10^6^ to 1.7 × 10^8^ cm^−2^ s^−1^ at 10,000 km, providing a total source rate to the magnetosphere of 2.1 × 10^26^ to 7.5 × 10^27^ s^−1^, for a source rate between 0.35 kg/s and 1.25 kg/s.

Unfortunately, there are no observational constraints with which to compare these model results. In this paper we report the detection of cold plasma in Saturn's magnetotail lobes, consider the interpretation of polar wind outflow, and use these observations to constrain the ionosphere as a source of plasma for Saturn's magnetosphere. In section 2 we describe the instrumentation used for this study; in section 3 we show an overview of the observations, the spacecraft trajectory, and the inferred upstream solar wind conditions. The detailed case study is presented in section 4 and various interpretations discussed in section 5. The implications for the physics of Saturn's magnetosphere are presented in section 5.

## 2 Intrumentation

We use data from the Cassini Dual Technique Magnetometer (MAG) [*Dougherty et al.*, 2004], the Cassini Plasma Spectrometer (CAPS) [*Young et al.*, 2004], the Magnetospheric Imaging Instrument (MIMI) [*Krimigis et al.*, 2004], the Radio and Plasma Wave Science instrument (RPWS) [*Gurnett et al.*, 2004], and the Ultraviolet Imaging Spectrometer (UVIS) [*Esposito et al.*, 2004].

CAPS measures the energy per charge and arrival direction of electrons and ions. The instrument consists of three sensors: the Electron Spectrometer (ELS) which measures electrons from 0.7 eV/*q* to 29 keV/*q*, the Ion Beam Spectrometer (IBS) which measures narrow ion beams from 1 eV/*q* to 50 keV/*q*, and the Ion Mass Spectrometer (IMS) that measures ions from 1 eV/*q* to 50 keV/*q*, followed by a time‐of‐flight (TOF) analyzer for the determination of mass per charge of incoming particles. A motor‐driven actuator rotates the sensor package to provide 208° scanning in the azimuth of the spacecraft, nearly 2*π* sr of the sky can be swept across every 3 min; spacecraft rolls can occasionally increase the field of view to 4*π* sr.

MAG measures the strength and direction of the magnetic field around Saturn via a fluxgate magnetometer (FGM) and a vector helium magnetometer (VHM) mounted on an 11 m spacecraft boom, with the FGM located in the middle of the boom and the VHM at the end. The magnetometer boom distances the sensors from the stray magnetic field associated with the spacecraft and its subsystems and, especially with spacecraft‐generated field variations, spacing the sensors at different distances along the boom allows this field to be better characterized and removed from the observations. This study uses data from the fluxgate magnetometer.

MIMI consists of three detectors: Charged Energy Mass Spectrometer (CHEMS), the Low Energy Magnetospheric Measurement System (LEMMS), and the Ion and Neutral Camera (INCA). CHEMS measures charge and compositions of ions with energy range between ≃3 to 220 keV/*q*, combining electrostatic deflection and TOF to measure the energy and composition of the energetic particles. INCA operates in two different modes, over the energy range between 7 keV/nucleon and 3 MeV/nucleon. In its ion mode INCA measures directional distribution, energy spectra, and composition of ions, and in its neutral mode, it takes remote images of the global distribution of the energetic neutral atoms, determining their composition and energy spectra for each image pixel. INCA has a field of view of 120° in latitude and 90° in azimuth, whereas when the spacecraft is rotating the camera covers about 4*π* sr. LEMMS consists of two oppositely directed telescopes, a low‐energy telescope designed to detect ions with energy ≥ 30 keV and electrons with energy between 15 keV and 1 MeV, and a high‐energy telescope for ions with energy range between 1.5 and 160 MeV/nucleon and electrons (0.1–5 MeV).

RPWS measures radio emissions, plasma waves, thermal plasma, and dust in the vicinity of Saturn. Three nearly orthogonal electric field antennas detect electric fields over a frequency range from 1 Hz to 16 MHz, and three orthogonal search coil antennas measure magnetic fields between 1 Hz and 12 kHz. A Langmuir probe is used to measure the electron density and temperature. Five receiver systems process signals from the electric and magnetic antennas.

UVIS measures ultraviolet light between the wavelengths of 55.8 and 190 nm for imaging spectroscopy and spectroscopic measurements of the structure and composition of the atmospheres of Titan and Saturn, rings, and surfaces, through two telescopes. It comprises two spectrographic channels: an extreme ultraviolet channel (EUV) that measures spectra between 55.8 and 118 nm and a far ultraviolet channel (FUV), which measures spectra between 110 and 190 nm.

## 3 Overview and Upstream Conditions

**Figure 1:**
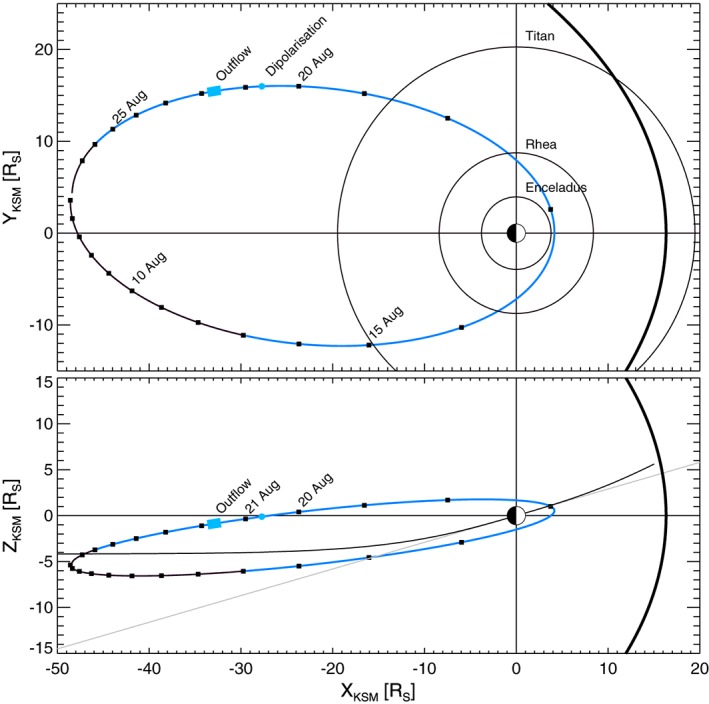
Cassini's trajectory in Kronocentric Solar Magnetospheric (KSM) coordinates for Cassini's revolution 27 of Saturn from 4 August to 28 August 2006 projected in (top) the *X*‐*Y* plane and (bottom) noon‐midnight meridional plane. The segment of the trajectory colored in thin blue lines indicates the time when Saturn was immersed in a solar wind compression region. The dot indicates the dipolarization observed on 20 August, and the case study period is indicated by the thick blue line. The magnetopause is shown as the thick black curve in both panels. In Figure 1 (bottom) the gray line indicates Saturn's equator and the curved black line the average location of Saturn's current sheet.

Figure 1 shows Cassini's trajectory on our day of interest, 21 August 2006 (day of year 233). The spacecraft was located in the dusk flank, about 36 *R*_*S*_ from the planet, north of the equator at ≃ 13.3° latitude, and in the premidnight sector at 22:13 local time. Cassini was on the outbound leg of revolution (orbit) 27.

**Figure 2:**
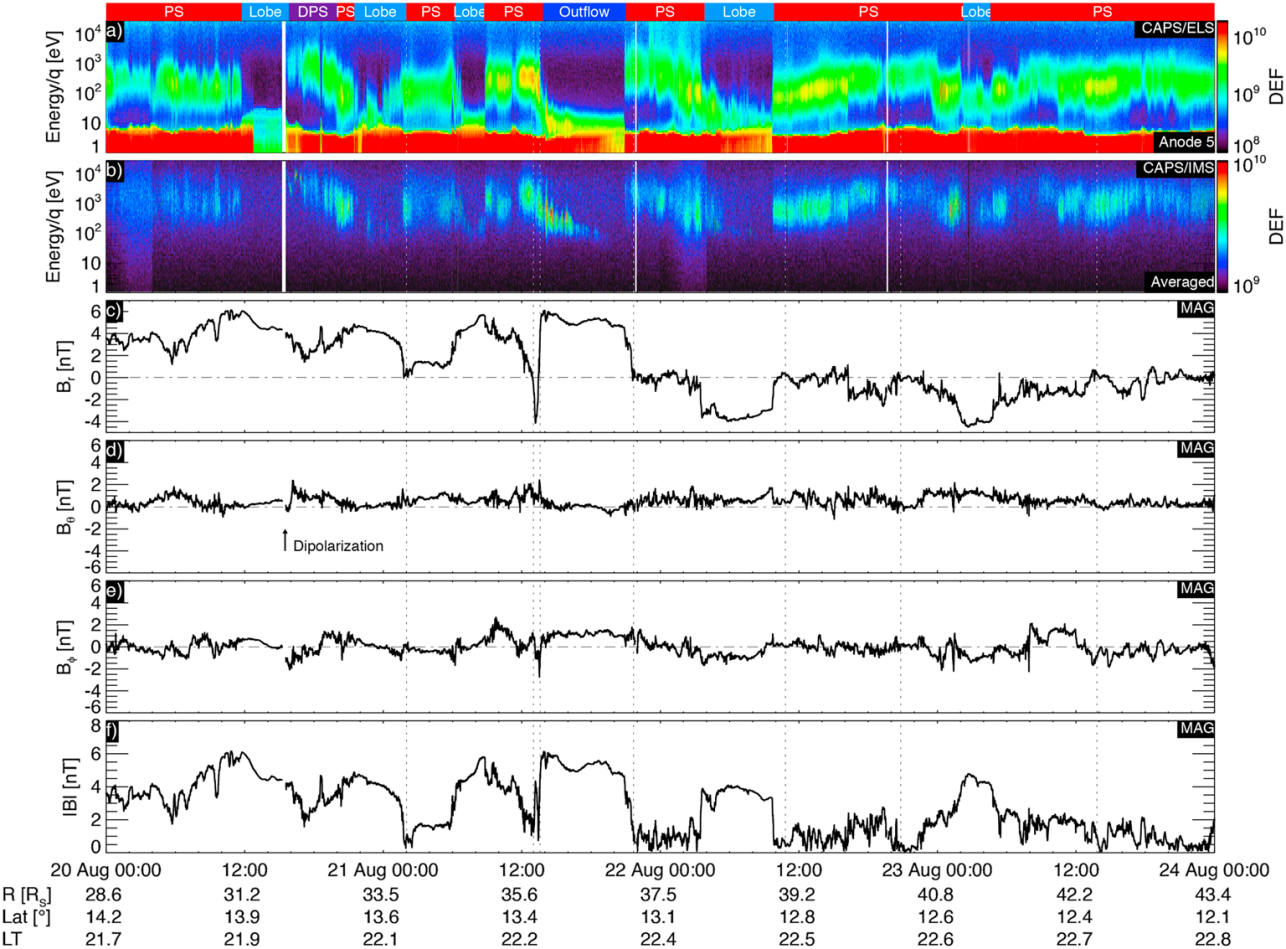
Overview of the interval studied in this paper, (a) ELS data from anode 5; (b) IMS data averaged over all anodes; both Figures 2a and 2b are in differential energy flux units (DEF) (m^−2^ s^−1^ sr^−1^ eV eV^−1^); (c–e) magnetic field components in KRTP coordinates *B*_*r*_, *B*_*θ*_, and *B*_*ϕ*_; and (f) |**B**|. Intervals when the spacecraft is in the lobes, and plasma sheet are highlighted at the top of the plot. Dotted lines indicate plasma sheet crossings. The arrow indicates the dipolarization observed on 20 August and discussed in *Jackman et al.* [2015]. The disturbed plasma sheet following the dipolarization is labeled “DPS” at the top, and the outflow event discussed in section 4 is highlighted as “Outflow.”

In Figure 2 we show time‐energy electron and ion spectrograms, and magnetic field components in the KRTP (Kronocentric Radial‐Theta‐Phi) coordinate system plus the field magnitude, for the time interval from 20 to 23 August 2006 (day of year 232–235). Both electron and ion spectrogram are represented in differential energy flux units (DEF) (m^−2^ s^−1^ sr^−1^ eV eV^−1^). IMS measures energy/*q* of incoming ions—hence, ions with same energy/*q* are recorded in the same bin—but the instrument has different response functions for different ion species. However, the best calibration available at the moment is the one that considers all the ion population made of protons. In KRTP coordinate system the *B*_*r*_ component of the magnetic field is positive pointing outward from Saturn, hence positive when the spacecraft is northward of the center of the current sheet, *B*_*θ*_ is positive pointing southward, and *B*_*ϕ*_ is positive in the corotation direction.

The colored boxes indicate when the spacecraft was located in various regions as determined from the magnetic field and plasma data. For example, the lobes are characterized by a strong and steady magnetic field, almost entirely in the *B*_*r*_ and *B*_*ϕ*_ directions, lack of both energetic particles and 100 eV plasma electrons. Centrifugal forces confine plasma to the equatorial region in giant planet magnetospheres. Since the field lines in the tail extend for long distances, the lack of thermal plasma on these tail field lines does not necessarily mean that the field lines are open: it may simply mean that the spacecraft is sufficiently far from the equatorially confined plasma that it cannot be detected. Current sheet crossings and encounters are identified with vertical dashed lines. The arrow in Figure 2d indicates a dipolarization event studied by *Jackman et al.* [2015]. Apart from the current sheet encounters and crossings, the radial component of the field is generally positive, until 22 August when it tends to be more negative, suggesting that typically, the spacecraft was north of the mean current sheet location until 22 August. The azimuthal field is close to zero but fluctuates, sometimes indicating a significantly swept‐forward field (*B*_*r*_ and *B*_*ϕ*_ having same sign) but sometimes swept back as it is over the rest of the Saturnian magnetosphere [*Vasyliuñas*, 1983]. *B*_*θ*_ is generally positive suggesting closed field lines.

The first period in the lobe is preceeded by the passage of a plasmoid at 1001 on 20 August 2006 and shortly after a data gap, from 1515 to ≃1530, is followed by a dipolarization at 1610 UT suggesting an extended interval of tail driving and subsequent relaxation [*Jackman et al.*, 2015]. Between 1530 and 1800 UT following the dipolarization, the plasma sheet is disturbed with an electron energy about 600 eV and fast directional planetward flow between 1 and 10 keV/*q*. Following this period the electrons and ions slowly reduce in energy, and hence, Cassini detects a cooler, more typical plasma sheet. During this period the magnetic field is swept forward.

The following four periods in the lobes are characterized by low‐energy ions and electrons, where the electrons are found just above the population of trapped spacecraft photoelectrons, sometimes almost indistinguishable from the spacecraft photoelectrons (around 10 eV). In each case the surrounding plasma sheet has electron energies typically found in the tail plasma sheet [*Arridge et al.*, 2009]. In the third lobe period during 1200–1800 on 21 August the electrons reach very low energies and appeared to be dispersed in time with lower electron and ion energies observed toward the end of the period in the lobe. During each of these four lobe periods the magnetic field is either purely radial or is significantly swept forward. After 23 August 2006 the plasma sheet and lobe period structure returns to that typically found in the magnetotail [*Arridge et al.*, 2009].

There is no upstream solar wind monitor at Saturn, so models and propagations from 1 AU are often used to infer upstream solar wind conditions [e.g., *Zieger and Hansen*, 2008; *Hsu et al.*, 2013; *Badman et al.*, 2015; *Baker et al.*, 2009; *Jasinski et al.*, 2014]. Propagation models, e.g., mSWiM [*Zieger and Hansen*, 2008], which propagate solar wind conditions measured at 1 AU, cannot be used for this interval since Saturn is far from apparent opposition during this period. In this work we use the ENLIL model, which is a time‐dependent 3‐D MHD heliospheric model [*Odstrcil et al.*, 2004] operated at the Community Coordinated Modeling Center at NASA Goddard Space Flight Center. This is the only heliospheric model that simulates solar wind conditions beyond 5 AU. ENLIL simulates supersonic, low *β* plasmas and must have inner coronal boundary conditions provided by either the Wang‐Sheeley‐Arge (WSA) [*Arge and Pizzo*, 2000] (inner boundary located at 21.5 solar radii) or MHD‐Around‐a‐Sphere (MAS) [*Riley et al.*, 2001] (inner boundary located at 30 solar radii) models. The outer boundary can be chosen to extend up to 10 AU as appropriate for simulations for Saturn.

**Figure 3:**
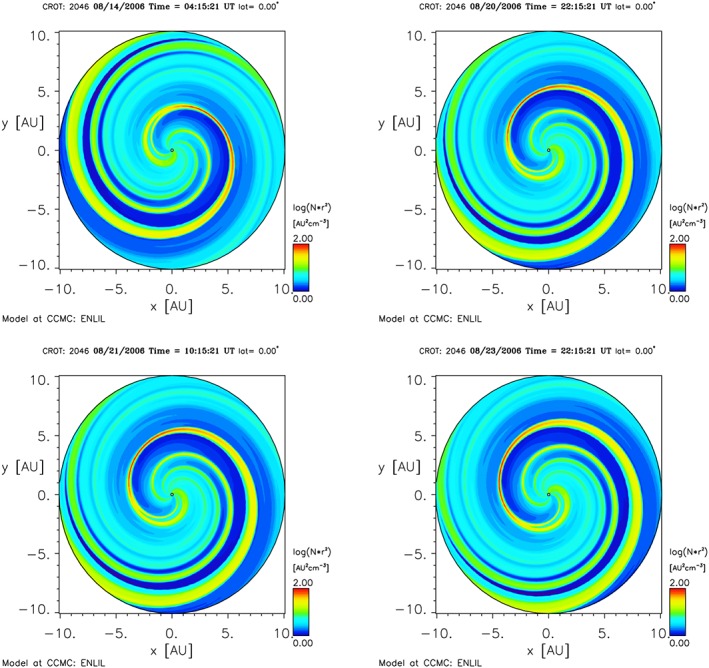
Simulated views of the plasma density (scaled with distance) in the ecliptic plane out to 10 AU. Saturn is near (−10,0) on the left‐hand side of these figures. These compare with various times surrounding the compression identified in Figure 4 and show the increases in solar wind dynamic pressure as the solar wind compression region rotates over Saturn.

**Figure 4:**
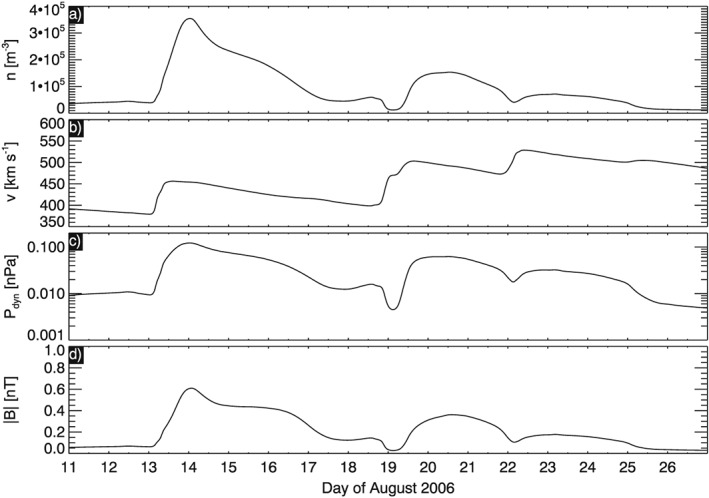
ENLIL simulation results for Carrington Rotation 2046 extracted at Saturn and plotted from 11 August to 27 August 2006. (a) Solar wind plasma number density, (b) solar wind speed, (c) solar wind dynamic pressure, and (d) the magnetic field strength in the solar wind.

ENLIL was run using Carrington Rotation 2046 as appropriate for this interval. Figure 3 shows the global heliosphere simulation during this period. In Figure 3 we can see density scaled with *r* ^2^, where *r* is heliocentric distance, from the heliosphere model. This shows a sequence of compression regions passing over Saturn. In Figure 4 we show a time series of solar wind conditions extracted at Saturn. We can compare the times of high solar wind density shown in Figure 3 with what we see in Figure 4, namely, solar wind density, speed, dynamic pressure, and total magnetic field strength in radial‐tangential‐normal coordinates (RTN). These show that this event is included in a solar event compression period. *Jian et al.* [2011] presented comparisons between ENLIL and Ulysses data at 5 AU and showed that the ENLIL predictions for the arrival of solar wind structures had an error of approximately 2 days. Even if this error is doubled to 4 days at 10 AU, Saturn is still immersed in a compression region during this event.

**Figure 5:**
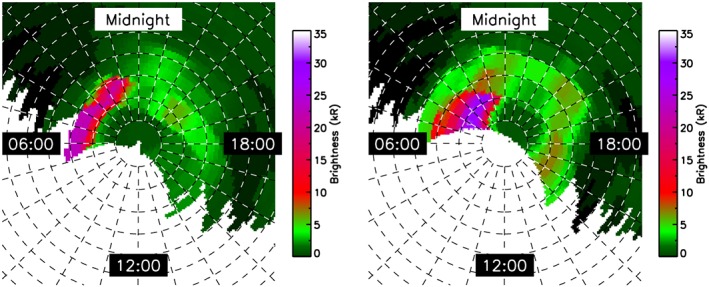
UVIS data for two periods before Cassini moves into the northern lobe from 05:40:16 and from 10:24:06 for about 4 h of data. The UVIS images are projected onto a colatitude‐local time grid where local noon is to the bottom of each figure and dusk to the right. The color scale indicates the intensity of the emission in kR.

We also studied Cassini remote sensing data to check if there were increases in activity of Saturn Kilometric Radiation (SKR) emissions and auroras that could support the simulation results, which suggest that Saturn is immersed in a solar wind compression region [Desch and Rucker, 1983; *Kurth et al.*, 2005; *Badman et al.*, 2008a; *Clarke et al.*, 2009; *Kurth et al.*, 2013]. It has to be considered, anyway, that *Stallard et al.* [2012] showed a delay of ≃8 h between the arrival of a solar wind compression and brightening of the aurora. In Figure 5 we show two auroral images taken by the UVIS instrument on Cassini. The data are projected onto a latitude and local time grid at 1000 km altitude: the figure shows the total FUV intensity, which is predominantly H and H_2_ emissions. Unfortunately, since Cassini is far from the planet and close to the equatorial plane, the view of the polar region is only partial and at low spatial resolution. Figure 5a shows a bright aurora, seen on the dawnside from 0030 to 0700 local time (with no viewing beyond 0700) of the Northern Hemisphere. The aurora reaches 30 kR between 0100 and 0700 local time from 12° to about 16° colatitude. Figure 5b shows a more extended aurora: we have two areas, one from midnight to 0600 local time, from about 4° to about 20°, with a brightness between 10 and 30 kR and a second area from 1500 to 1900 local time, from 8° to about 14°, with a brightness that reaches ≃ 7 kR. Clarke et al. [2009] reports similar brightness for aurora during disturbed conditions. Since Cassini is far away from Saturn, orbiting in the equatorial plane, the auroral emissions observed by UVIS are subject to significant limb brightening, whereby the emissions are viewed through a long column of atmosphere near the poles, compared to lower latitudes. This was corrected using the sine of the emission angle, but since each UVIS pixel covers a large area on the planet, the images could still be partially affected by the limb brightening, which, however, does not affect the extension of the aurora or the presence of aurora itself. These auroral emissions, the fact that the aurora is extending poleward and is brighter on the dawnside, suggest that there are some tail dynamics influencing the auroral region and which has been shown to be a consequence of the passage of solar wind compression regions [Stallard et al., 2008; Cowley et al., 2005].

**Figure 6:**
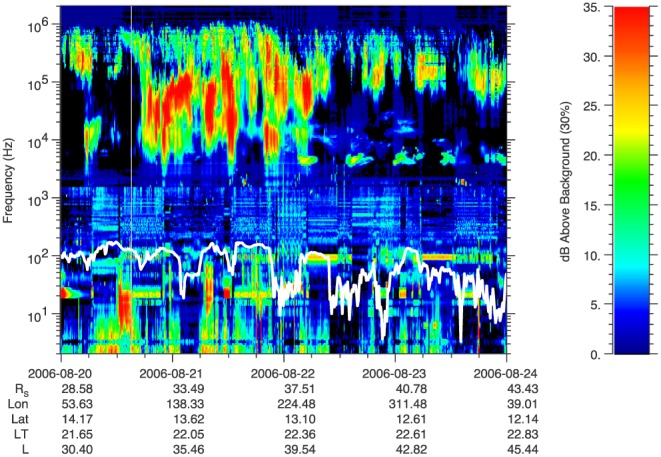
RPWS electric field data from 20 August to 23 August 2006 showing the received power relative to the background with the electron cyclotron frequency (calculated from the magnetic field strength) overlaid in white.

Figure 6 shows electric field spectrogram, up to 2 MHz, from RPWS. In this time range the emissions above 3 kHz are Saturn Kilometric Radiation (SKR) and extend until 0600 UT on 22 August with low‐frequency extensions [*Jackman et al.*, 2009]. After this time, narrowband periodic emissions are observed near 5 kHz that are probably generated closer to the planet, hence not likely to be associated with plasma detected near the spacecraft. Narrowband emissions are also observed around 2 kHz, notably at 1530 on 21 August and at 1430 on 23 August, possibly associated with electron plasma oscillations. These can be used to infer the electron density from the frequency which suggests a density of 0.05 cm^−3^, compatible with the CAPS/ELS electron moments. The spectrum below 1.5 kHz is noisy, mostly probably given by interference from the spacecraft reaction wheels. However, below about 50 Hz, there are quite visible features (middle of 20 August and just after 06:00 on 21 August) not generated by spacecraft interference. An examination of the corresponding magnetic spectrogram (not shown) shows that these features do not have a magnetic component.

More diffuse broadband emissions below 10 Hz might be associated with ionospheric outflow and are seen to correlate with the observation of cold plasma in the tail lobes as identified in Figure 2. These emissions may be whistler mode emissions as detected in the magnetotail of Uranus [*Kurth et al.*, 1989]. However, the corresponding magnetic field spectrogram (not shown) does not show a magnetic component to this diffuse broadband emission, suggesting an electrostatic mode.

The SKR observations in Figure 6 show evidence of a brightening in SKR near 1800 UT on 20 August as noted by *Jackman et al.* [2015] and which may be associated with the dipolarization event at 1610 UT reported in that study. SKR emissions are active throughout the rest of the 20 August and 21 August, appearing to switch off early on 22 August, clearly showing evidence of magnetospheric dynamics during this period [Desch, 1982; *Kurth et al.*, 2005; *Badman et al.*, 2008b; *Jackman et al.*, 2009]. The SKR main spectrum, which typically ranges between 100 and 400 kHz, is generated by the cyclotron maser instability. This is in contrast to the lower frequency narrowband emissions mentioned in the previous paragraph which are likely caused by a different mechanism altogether.

## 4 Data Analysis

**Figure 7:**
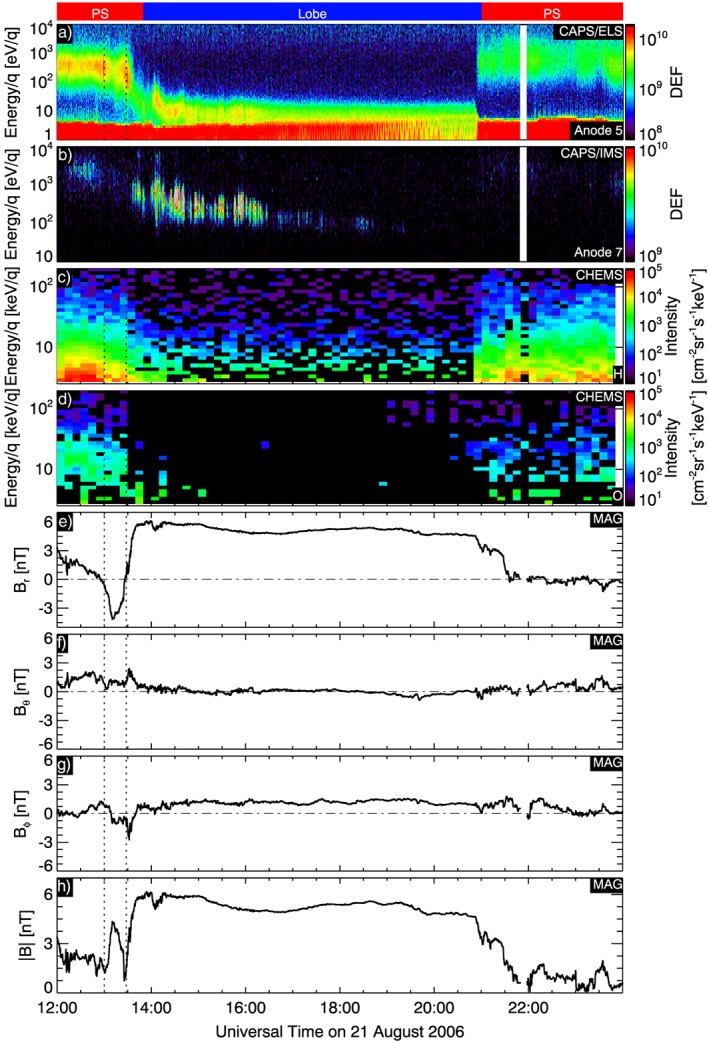
Interval studied in this paper, (a) ELS in anode 5, (b) IMS data from anode 7. Both Figures 7a and 7b are in differential energy flux units (DEF) (m^−2^ s^−1^ sr^−1^ eV eV^−1^); (c) CHEMS energetic H^+^ spectrogram, (d) CHEMS energetic W^+^ spectrogram, (e–g) magnetic field components in KRTP coordinates *B*_*r*_, *B*_*θ*_, *B*_*ϕ*_, and (h) |**B**|. The two dotted lines indicates the two plasma sheet crossing before the spacecraft moves in the northern lobe.

Turning our attention to the specific time interval that we focus on in this case study. Figure 7 is a zoom‐in of the case study interval, from 1200 to 2400 UT on 21 August, from Figure 2, and shows the characteristics of this event from different instruments on Cassini.

**Figure 8:**
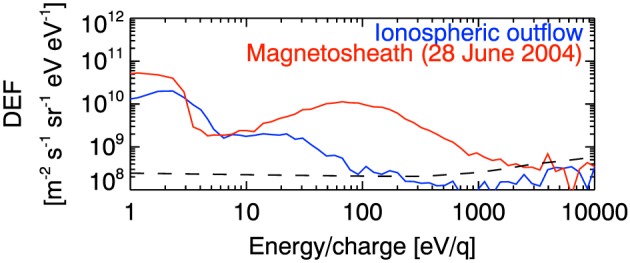
Comparison between two electron spectra: electron spectrum from this case study, 21 August 2006, 1400 UT (blue line), electron spectrum from 28 June 2004, 1400 UT, a typical magnetosheath spectrum (red line), with superimposed (dashed line) the one count level. Both spectra are from anode 5 in Cassini ELS. The magnetosheath population seen below ≃ 3 eV are photoelectrons, whereas in our case study we find photoelectrons below ≃ 8 eV.

Looking at the electron distributions first (Figure 7a), we see that at 1330 on the 21 August (when the spacecraft moves completely into the northern lobe) the population below around 5 eV are trapped spacecraft photoelectrons and the upper edge of this distribution shows that the spacecraft potential is around 5 V. Typically, the potential in the lobes is 30–50 V, so this is consistent with the presence of dense plasma in the lobes. From 1330 UT the ambient electron energy drops from 10^2^ eV to a few eV, and this ambient population is sometimes hard to distinguish from the trapped spacecraft photoelectron distribution, especially toward the end of the interval. Further evidence of the unique nature of this event is revealed by the low energy of these electrons since in the quiet tail lobes, *Arridge et al.* [2009] finds an electron temperature of ≃100 eV.  Moreover, Figure 8, where we compare two electron spectra, one from this event and one from the magnetosheath, shows how colder the electron population for this event is compared to another region of the magnetosphere.

**Figure 9:**
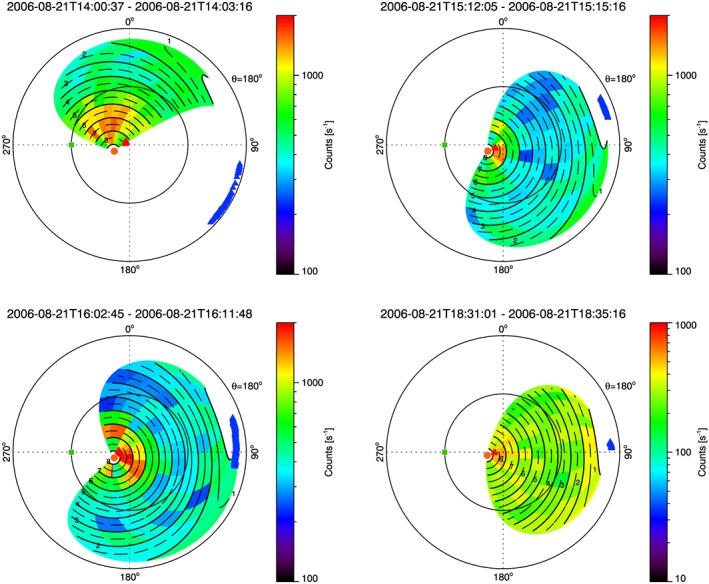
Measured ion counts as a function of look direction about the spacecraft for four different times in the northern lobe. In each case ions are observed flowing tailward from the direction near Saturn. The energy/*q* ranges are, respectively, from left to right, from top to bottom, 181.1–2048, 107.7–724.1, 107.7–724.1, 90.40–361.9 eV/*q*.

Figure 7b show the ion populations with ≃ 3 keV/*q* ions in the plasma sheet and lower energies in the lobe. The measured ion fluxes are larger than in the plasma sheet and are also seen to slowly disperse in energy from ≃ 500 eV/*q* to ≃ 100 eV/*q* over a period of around 4 h. During the period in the plasma sheet, the field of view of IMS does not cover the ideal corotation direction and sees only weak fluxes from directions > 30° from corotation. However, during the period in the lobes, IMS views flows coming from the direction of Saturn. Figure 9 shows measured ion fluxes as a function of the look direction around the spacecraft, in a polar projection, expressed in OAS coordinate. In this coordinates system **S** is the axis along the Cassini‐to‐Saturn line, **O** is defined by **S**× (*Ω* ×**S**), where *Ω* is the planet spin axis and **A** completes the right‐hand system. We can represent a point around the spacecraft with two angles relative to the **S** axis: *θ* (range from 0° to 180°) is the latitude angle, so it is the polar angle away from Saturn, and *ϕ* (range from 0° to 360°) is the azimuth around **S** axis, referenced to 0° in the **O** direction. Specifically, in Figure 9, *θ* = 90° is represented by the inner circle and *θ* = 180° is the outer circle. Hence, these plots show the presence of a cold ion population with a width of ≈40° flowing tailward.

The ion composition during this interval is also unusual and was determined by a fit of CAPS/IMS time‐of‐flight data to a forward model [e.g., *Thomsen et al.*, 2010]. In the plasma sheet between 1200 and 1320, where Cassini crosses the plasma sheet twice, passing from the north lobe to the south lobe, and coming back to the north lobe again, H^+^ counts are ≃10^4^ and (*m*/*q*= 2) ≃ 10^3^, and the ratio of water group ions to hydrogen, [W^+^]/[H^+^], and *m*/*q*= 2 to hydrogen, [*m*/*q*= 2]/[H^+^], are 1.79 ± 1.58% and 2.45 ± 0.15% respectively. Hence, the plasma sheet appears to be devoid of water group ions. After 1320, once the spacecraft is in the north lobe, H^+^ counts are ≃10^5^, 1 order of magnitude larger than the counts of when the spacecraft was crossing the plasma sheet and *m*/*q*= 2 counts are 5 times larger. During this time period, the ratio between water group ions and hydrogen [W^+^]/[H^+^] is zero within error and [*m*/*q*= 2]/[H^+^] = 2.23 ± 0.04%. From 2130 to 2400, the spacecraft returns to the plasma sheet, H^+^ counts are ≃10^4^, 1 order of magnitude lower than in the lobes and *m*/*q*= 2 counts diminish to 10^2^. During this time period the ratio between water group ions and hydrogen [W^+^]/[H^+^] is again zero within error and [*m*/*q*= 2]/[H^+^] = 3.22 ± 0.29%.

We checked previous and following spacecraft orbits at the same latitude and the same local time. For the previous orbit (28 July 2006), the lobes are empty of ions and when the spacecraft crosses the plasma sheet twice between 0400 and 1200, [W^+^]/[H^+^] ≃ 30.02 ± 15.11% and [*m*/*q*= 2]/[H^+^] = 24.79 ± 0.26%. On the following orbit (13–14 September 2006) the spacecraft seems located always in the lobes, which are mostly empty of ions. Therefore, we consider this an atypical time interval.

Looking at MIMI/CHEMS data in Figures 7c and 7d, we find that between 1400 and 2030, the lobes are populated by hot H^+^ with foreground.  Hot O^+^ ions start to appear at around 18:45, and they seem slightly dispersed in energy.  Higher intensities are observed between 100 and 300 keV, while INCA sees O^+^ even for larger than 500 keV (see next paragraph). The pitch angle distribution for this population is generally between 30 and 90°, implying an outward flow.

**Figure 10:**
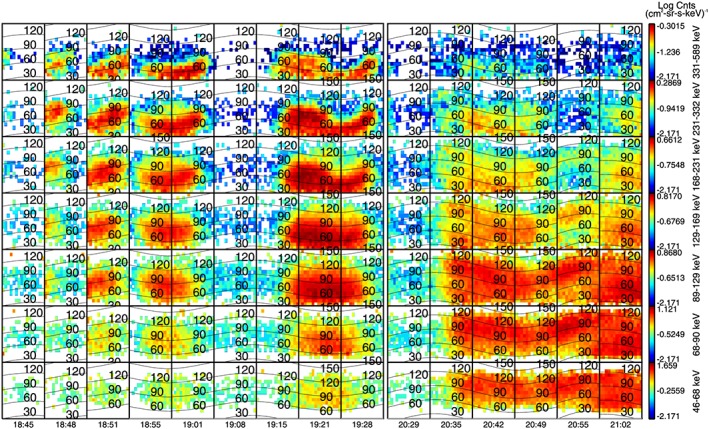
MIMI/INCA data: O^+^ with energies between 46 and 589 keV. The overlapped black contours represent different pitch angles values.

Throughout this interval the MIMI/INCA camera is in ion mode and so provides additional information on the energetic ions. When the spacecraft is in the lobes, we find no O^+^ during most of the interval. Figure 10 shows O^+^ distributions observed by INCA from 18:45 to 19:28 and from 20:29 to 21:02.

By looking at the INCA look direction and the pitch angle coverage in the columns corresponding to these intervals, we know that during this time, the spacecraft orientation is steady. Afterward, we see energy peaks periodically between 18:55 and 19:01, 19:15 and 19:28, 19:42 and 19:55, and 20:08 and 20:22, associated with a first‐order anisotropy, when the spacecraft starts rolling: an entire rotation is enclosed by approximately four white squares corresponding to the period in the intensity peaks [e.g., *Kane et al.*, 2008]. The pitch angle distribution is peaked between 0° and 90° indicating ions flowing downtail, with scattering accounting for the intensities that appear between 90° and 120°. The highest flux is detected for energies between 89 keV and 589 keV and a very low flux for lower energies, until 20:35, when the O^+^ covers energies from 46 and 589 keV. The gyroradius for 89 keV to 589 keV ions is ≃ 0.6 to 1.5 *R*_*S*_;  hence, we interpret the ions before 20:35 as a remote detection of the plasma sheet while the spacecraft is the lobes. After this point the spacecraft approaches the plasma sheet (around 20:55), and we see O^+^ of all energies in the detector; the flow has a broader pitch angle distribution, which is more focused between 0° and 120° with increasing energy. Observing what happens to the magnetic field at the same time, we notice that the magnetic field has lowered, showing a step of about 0.5 nT before 2000 in *B*_*r*_ and *B*_tot_ trend, then a bigger drop in the field of more than 1 nT.  Finally, at 2122 UT, the flux seems to be isotropic and the magnetic field reaches low field strengths, indicating the plasma sheet encounter.

INCA observations of H^+^ are contaminated with O^+^ due to an instrumental effect but are consistent with isotropic H^+^ in the lobes and an increasing flux of H^+^ in the plasma sheet toward the end of the interval.

The magnetic field is swept forward during this all interval,  namely,  *B*_*r*_ and *B*_*ϕ*_ components of the field maintain the same sign for more than 8 h. With a single spacecraft it is difficult to separate spatial and temporal effects and it is possible that this swept‐forward field configuration was a characteristic of this local time in Saturn's magnetosphere. Many of Cassini's orbits have nearly identical coverage in local time and latitude, so to check if the field is typically swept forward at this radial distance and local time, we examined the sweep‐back angle during the orbits of Cassini before (28 July 2006) and after (13 and 14 September 2006) the orbit during this case study. The spiral angle of the field indicated that although the field was generally almost meridional (not swept forward or swept back) during these orbits, only this orbit had the swept‐forward configuration indicating an unusual configuration.

*Jackman and Arridge* [2011] studied the magnetic field strength in the lobes and established the average field strength at various radial distances fitting this to a power law function of radial distance, *r*, in units of *R*_*S*_, such that *B*_lobe_(nT) = (251 ± 22)*r* ^−1.2 ± 0.03^. At 36 *R*_*S*_ this expression predicts a field strength of 3.4 ± 0.3 nT which is 2 nT smaller than observed, indicating either a highly compressed magnetosphere or where the magnetotail was loaded with open magnetic flux, or both.

## 5 Interpretation and Discussion

In interpreting these observations we have considered several possibilities for the presence of cold ion beams at large distances in Saturn's magnetotail. Magnetic reconnection would result in rapid ion flows in the range 144–1240 km/s (≃ 10 keV) [*Hill et al.*, 2008; *Jackman et al.*, 2014] and energized electrons in the beam with planetward and tailward ion flows depending on location relative to the X line. In this case no such energized electrons are observed, the observed ion energies are small, and no large *B*_*θ*_ deflections are observed.

In the Saturn system cold plasma usually originates from ionization in the inner magnetosphere. These cold plasma observations could potentially be the result of rapid cold plasma transport from the inner magnetosphere. In this case, however, we would detect water group ions from the moons and we would expect the ions to be centrifugally confined. Furthermore, in our observations we find (*W*_⊥_/*B*≃ 2 eV/nT) and conservation of the first adiabatic invariant implies that we should find a similar ratio close to the source of this ion population. According to *Arridge et al.* [2011, and references therein], who synthesized the results of many studies, we see that at distance of 5 *R*_*S*_, 8.7 *R*_*S*_, and 20 *R*_*S*_, we would find, respectively, a *W*_⊥_/*B*≃ 0.005, *W*_⊥_/*B*≃ 0.3 and *W*_⊥_/*B*≃ 9 eV/nT. Hence, we do not find a ratio ≃ 2 close to the planet. Furthermore, the composition is quite different to what is usually seen in the inner and middle magnetosphere. It is also difficult to envisage a physical mechanism for removing ions from the inner magnetosphere to the tail in the form of a narrow directional ion population.

Finally, an alternative interpretation is that we detect Saturn's plasma mantle: ions that have entered the dayside magnetosphere via dayside reconnection and have mirrored and flowed out tailward into the magnetotail. We would expect a solar wind plasma composition of [*m*/*q*= 2]/[H^+^], namely, ≃4% which is similar to 2.23 ± 0.04% from time‐of‐flight fits. We would also expect the particles to conserve the first adiabatic invariant between the magnetotail and the cusp. From *Jasinski et al.* [2014] the electron temperature in the cusp is ≃40 eV in a field strength of 8 nT; thus, *W*_⊥_/*B*≃ 5 eV/nT, so we would expect electron energies in the magnetotail to be 25 eV, much higher than observed. At Earth, the electrons in the mantle have same energy as the electrons in the magnetosheath [*Formisano*, 1980], and we would expect the same to happen at Saturn. Figure 8 shows that the electron population for this event is significantly colder than the electron population in Saturn magnetosheath.

Furthermore, we can also examine the convection timescale for newly opened flux tubes compared with the speed of the ions. Assuming a flux tube moves tailward at 40 km/s (≃10% of the solar wind speed), it would take 16.7 h to traverse the 40 *R*_*S*_ from the dayside to the magnetotail. H^+^ with 1 keV energy would have covered a distance of 436 *R*_*S*_ in the same time, and the 50 eV H^+^ a distance of about 100 *R*_*S*_. This suggests that by the time the flux tube travels from dayside to the spacecraft position, it would be already emptied of ions. If we considered the rotation time instead (about 5 h), we would find that 1 keV and 50 eV H^+^ would travel, respectively, to distances of 131 *R*_*S*_ and 29 *R*_*S*_. This leads us to not consider valid a mantle provenance for the plasma in our event.

### 5.1 Ionospheric Outflow

For ionospheric outflow we would expect cold electrons and ions flowing tailward from Saturn into the magnetotail via the magnetotail lobes, as observed with CAPS/IMS and CAPS/ELS. We would expect the ion composition to be consistent with Saturn's ionosphere, i.e., H^+^, $H_{2}^{+}$, and $H_{3}^{+}$. In the CAPS data, the ions are dominated by H^+^ with a smaller contribution from a species with *m*/*q*= 2 which cannot be separated into $H_{2}^{+}$ and He^++^. Unfortunately, $H_{3}^{+}$ has a time of flight in CAPS/IMS which lies near an instrumental artifact and therefore cannot be extracted at this time. Hence, we interpret this event as ionospheric outflow via a polar wind.

It is not possible to determine the connectivity of field lines (open or closed) during the period of ionospheric outflow. This is a period of intense magnetospheric activity, and we think that precipitating electrons producing auroral emissions could happen simultaneously on the same field line as ionospheric outflow but still in an upward current region. Hence, the auroral emission and source for ionospheric outflow could be collocated in the same region of the ionosphere. *Bunce et al.* [2008], used Cassini and Hubble Space Telescope data to show that the southern auroral oval is located at the boundary between open and closed field lines. However, *Jinks et al.* [2014], using Cassini data, found that the poleward edge of the upward current region is displaced equatorward from the polar cap boundary in both the northern and southern hemispheres. Thus, the closed field line region can be present also beyond the upward current region poleward boundary. This could imply that the spacecraft was located on closed field lines.

**Figure 11:**
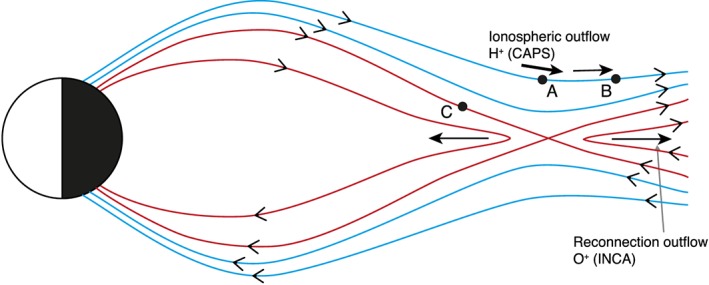
In the schematic we represent different field lines with different colors. In point A the spacecraft is in the north lobe, only detecting ionospheric ions in CAPS/IMS. The ionospheric ions are represented with arrows, which get thinner approaching point B, in order to represent the dispersion. When the spacecraft is located in B, from 1845, cold ions from ionosphere are still detected in CAPS/IMS but meantime MIMI/INCA remote senses hot O^+^ from the plasma sheet. O^+^ is flowing downtail accelerated by reconnection and is represented in the plot through an arrow. After 2100 the spacecraft returns to the plasma sheet (point C).

While in CAPS/IMS we see cold dispersed ions, we argue that the ions detected in MIMI/INCA and MIMI/CHEMS from 18:45 belong to the plasma sheet: these ions are flowing downtail at speeds ≃ 1000 km/s. The fact that the plasma sheet is emptied from *W* ^+^ in the range of CAPS/IMS might suggests that the plasma sheet has been emptied through a reconnection. In this scenario, while in the lobes CAPS/IMS detects ions flowing downtail coming from the ionosphere, MIMI/INCA is remotely sensing ions flowing downtail accelerated by reconnection. Hence, we think that reconnection is happening at the boundary between the lobe and the plasma sheet beneath the spacecraft, while the spacecraft is on open field lines (see Figure 11).

According to magnetospheric magnetic field models [*Khurana et al.*, 2006; *Bunce et al.*, 2003], this region of the magnetosphere is only slightly swept forward, but the sweep forward increases during periods of increased solar wind dynamic pressure. Since these models only include azimuthal fields due to magnetopause currents, this then shows that the swept‐forward configuration is due to magnetopause currents. The reason why we do not see a strongly swept forward configuration in the previous and following orbits, at about same latitude and same local time, is due to the corotating interaction region (CIR) that is passing the planet during this specific time period.

*Glocer et al.* [2007] coupled a polar wind outflow model with an MHD model of Saturn's magnetosphere to estimate the number flux of ions outflowing from Saturn's ionosphere in a steady state. They found value between 7.3 × 10^6^ and 1.7 × 10^8^ cm^−2^ s^−1^. To compare our observations with the *Glocer et al.* [2007] simulations, we estimated the number flux, *n* *v*, where *n* is the number density and *v* is the speed of ions in the tail, and used conservation of magnetic flux to scale these to their values closer to Saturn.

The generally low numbers of counts during this event make the ion moment calculations challenging, so we assumed that the ion number density was equal to the electron number density. The ion speeds were estimated by fitting the ion spectra with Gaussian plus a background. Fits were filtered using the *χ* ^2^ for each fit and a manual inspection of the fit. The fits were performed on the IMS anodes where peak fluxes were observed. The peak energy from this fit was taken as the ion bulk flow energy (actually an upper limit since this assumes that the ions are completely cold). The ion speed was found to be ≃400 km s^−1^ at about 1340, with the speed slowly diminishing to get to ≃200 km s^−1^ at about 1700 UT.

**Figure 12:**
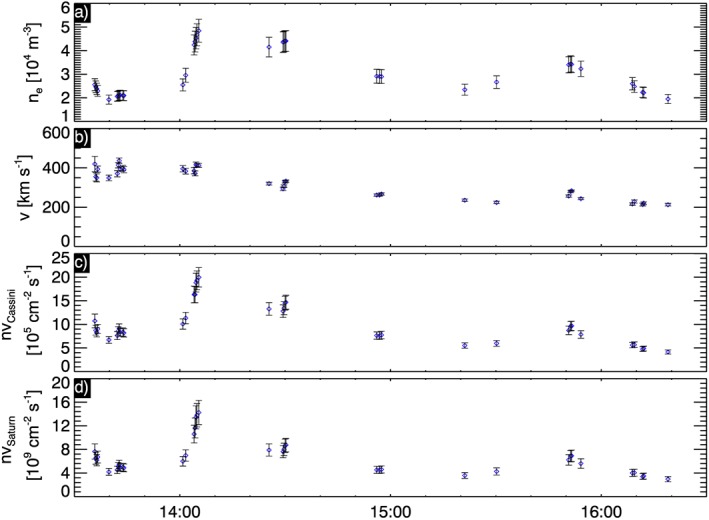
Estimates of the number flux associated with the polar wind. (a) The measured electron density at Cassini, (b) the estimated ion speed at Cassini, (c) the estimated number flux at Cassini, and (d) the number flux estimated at an altitude of 10,000 km above Saturn at 78° latitude.

Figure 12 shows the number density, speed, and calculated tail number flux, *n*_*t*_ *v*_*t*_. We assumed 10% uncertainty on the electron densities [*Arridge et al.*, 2009]; the speed uncertainties were obtained by propagating the uncertainties in the peak energies found from our nonlinear fits. The number flux uncertainties were calculated by propagating the uncertainties on *n*_*t*_ and *v*_*t*_.

*Glocer et al.* [2007] presented number fluxes at an altitude of 10,000 km. To map our observed number fluxes to this altitude, we assume that the number of outflowing ions are conserved in a flux tube from the ionosphere to the tail, and use *B*_*t*_ *A*_*t*_=*B*_*i*_ *A*_*i*_, and therefore scale the tail number flux to get the ionospheric number flux by *n*_*i*_ *v*_*i*_=*n*_*t*_ *v*_*t*_ *B*_*i*_/*B*_*t*_. The ionospheric field strength was calculated from a dipole at an altitude of 10,000 km at an auroral colatitude of 12°. Using the observed tail field strength shown in Figure 7h, we then calculate the ionospheric number fluxes as shown in Figure 12d. We obtained ionospheric number fluxes between (2.95 ± 0.43) × 10^9^ and (1.43 ± 0.21) × 10^10^ cm^−2^ s^−1^. These estimates are 1 order of magnitude larger than the value obtained by *Glocer et al.*[2007].

One possible interpretation for this discrepancy is due to the fact that the model was run for a steady atmosphere and steady magnetosphere, hence classical polar wind as defined in *Schunk* [2007]. We argued that this event occurred during a CIR (corotating interaction region) compression and with substantial magnetospheric activity, which produced enhanced outflows, namely, generalized polar wind, as defined in *Schunk* [2007]. Moreover, due to low counts, it is very difficult to evaluate the velocities with fits that also consider the temperatures. From a preliminary estimate, we think that the speed we calculated overestimate the velocities of a factor of about 2. This would affect the number flux of a factor of 2, which is a minor contamination for our estimate. In addition, *O'Donoghue et al.* [2015, and references therein] find a neutral temperature not higher than 650 K, which from *Glocer et al.* [2007] should lead us to higher values of number flux.

We calculated the total particle source rate using the polar cap area range extracted by *Glocer et al.* [2007], obtaining an estimate between 8.6 ×10^28^ and 6.3 ×10^29^ s^−1^, that leads to a mass source between 1.4 × 10^2^ and 1.1 ×10^3^ kg/s, if there were no loss in the tail.

By contrast, using the mean position of the northern and southern aurora (respectively, 15.1 ± 1.0° and 15.9 ± 1.9°) [*Carbary*, 2012], we can recalculate the area of the polar cap and we obtain instead a rate of 49.1 ±9.8 × 10^27^ and 23.7 ±4.7 × 10^28^ s^−1^ and a mass source between 82.0 ±16.5 and 395.6 ±79.2 kg/s.

If we considered instead, more realistically, an active area covering the region of auroral emission [northern and southern extending, respectively, between 13.4° and 16.8° and between 12.9° and 18.9°, average values taken from *Carbary* 2012], we would obtain a source rate between 29.7 ±8.1 × 10^27^ and 14.3 ±3.8 × 10^28^ s^−1^ (mass source between 49.7 ± 13.4 and 239.8 ± 64.8 kg/s).

Specifically, for our event, we can see, from UVIS data, the active area from auroral emissions in the hours prior to the event: it extends from 5° to 12°, from 0000 to 1900 LT, with a data gap from 0600 to 1430 LT. If we considered an outflow only from the brightest area (we then consider the area of the ring which includes the brightest area from dawn to dusk, divided by 2 to consider the area with no auroral emission and the data gap) with an estimated error of 2.5° (half of the projected polar grid resolution), we would obtain 6.1 ±2.9 × 10^27^ and 2.9 ±1.4 × 10^28^ s^−1^ for a total mass source of between 10 ±4 and 49 ± 23 kg/s.

Evaluating the fact that the whole auroral oval is not completely active in our event and the active area has a different brightness, so possibly the outflow is not coming out uniformly, a useful quantity to be defined is the rate and the mass source per hour of local time, spanning 5° in latitude (in our event mainly from 5° and 10°): 3.2 ±1.9 × 10^26^ and 1.5 ±0.9 × 10^27^ s^−1^ per hour of local time, hence between 0.5 ± 0.3 and 3 ±1.5 kg/s per hour of local time.

The mass source estimates for different active areas are here calculated only for the northern cap, the northern auroral, and the aurora in the north pole we remote sense with Cassini/UVIS. Moreover, the source rates calculated are upper limits of the ionospheric mass contribution to the magnetosphere: we do not have any estimate at the moment on how much of this mass stays indeed in the magnetosphere.

The ion dispersion can be generated by three different processes, two of them consisting in a temporal variation and one of them in a spatial variation. The first temporal effect is due to the fact that the energies of the particles emitted from the same area in the ionosphere have a Maxwellian distribution. Consequently, there is a velocity filter effect whereby faster ions reach the spacecraft before slower moving ions, producing a continuous velocity dispersion in the data. The second temporal scenario can be caused by a time variability of the source. Therefore, the spacecraft, in this case, would be detecting ions originated from the same source, but a source that was in an exited state at the beginning of the time interval, and then relaxed afterward, emitting lower energy ions toward the end of the interval. Lastly, the spatial scenario could be caused by the fact that the spacecraft moves across different field lines which have their feet in different latitudes in the ionosphere. At the beginning of the interval, the spacecraft would have been located on a field line connected to an intensely exited region of the ionosphere, and then the spacecraft moved onto field lines connected to a calmer region of the ionosphere. Unfortunately, from the data is not possible to distinguish among these three different scenarios. A combination of two or all of these processes may be involved in producing the observed ion energy dispersion.

We know that the presence of an electron dispersion in the data maybe produced by the same process that caused the ion dispersion.

If we interpret the ion dispersion as a velocity filter effect, whereby faster ions reach the spacecraft before slower moving ions from a spatially restricted source, then we can obtain the distance to that source from a time‐of‐flight expression *t* = *t*_0_+*d*/*v*, where *t* is the time of observation, *t*_0_ is the time at which all the ions left the source, *v* is the speed of an ion observed at time *t*, and *d* is the distance to the source. We used the velocities and times from Figure 12, calculated the inverse velocity, and then fitted a straight line to *t* as a function of 1/*v* to obtain the intercept (*t*_0_) and the gradient (*d*). Therefore, *d* is an estimate of the field‐aligned distance that the ions traveled and the time at which they left their source *t*_0_. The ion counts were not considered to be sufficiently far above background to perform this analysis using energy‐time spectra, and hence, we have performed the moment analysis as an alternative. The distance was found to be 69 ± 3 *R*_*S*_. Considering that our speeds are possibly overestimated by a factor of ≃ 2, we then obtain a distance of 34 ± 1 *R*_*S*_. This is consistent with the length of a field line from Cassini to Saturn's ionosphere calculated by tracing field lines in a magnetospheric field model [*Khurana et al.*, 2006], thus strengthening the interpretation of this event as ionospheric outflow.

## 6 Conclusions

We presented a case study of an event from Saturn's magnetotail from 21 August (day of year 233) 2006. The event is enclosed in a time period when the magnetosphere was compressed by a region of high solar wind dynamic pressure, as identified from a global heliosphere simulation (ENLIL) and auroral and SKR intensifications. Cold ions and electrons are detected in the lobes, and the cold plasma ion composition does not show evidence for *W* ^+^ ions, neither when the spacecraft is located in the plasma sheet or when Cassini is in the lobes.

After considering different interpretations, we conclude that this event is an example of ionospheric outflow in Saturn's magnetotail. This is the first time that a low‐energy ionospheric outflow event has been detected at planets other than Earth, helping understand how ionospheric outflow contributes to the magnetosphere. We estimate an ionospheric escape number flux (at an altitude of 10,000 km) between (2.95 ± 0.43) × 10^9^ and (1.43 ± 0.21) × 10^10^ cm^−2^ s^−1^ 1 or 2 orders of magnitude larger than the estimate obtained by *Glocer et al.* [2007], which represents a mass source between 1.4 ×10^2^ and 1.1 ×10^3^ kg/s. Furthermore, we estimated the mass source provided by the ionosphere, for different configurations of the active region at the northern pole. Considering the most probable scenario as an active region that covers the main auroral oval, we obtain a mass source between 49.7 ±13.4 and 239.8 ±64.8 kg/s, comparable with what was found for Enceladus (60–100 kg/s) [*Fleshman et al.*, 2013]. Specifically for the auroral morphology found during this event, we obtained a mass source of between 10 ±4 and 49 ±23 kg/s. However, we do not have any current estimate on how much of the mass provided by the ionosphere, stays indeed in the magnetosphere, and how much instead gets lost downtail. As such, our estimates are an upper limit to the magnetospheric mass source.

Future work will include a survey to search for evidence of other ionospheric outflow events at Saturn; besides, modeling ionospheric outflow from Saturn's ionosphere with a dynamic magnetosphere and atmosphere is needed to understand the relationship between reconnection or magnetosphere‐ionosphere coupling and the outflow from the atmosphere. Moreover, other interesting steps could involve an estimate on how much of the mass flowing in the tail from the ionosphere remains in the magnetosphere and how much is lost downtail. A search for evidence of low‐energy $H_{3}^{+}$ in the magnetosphere will also provide further evidence for ionospheric outflow. Cassini proximal orbits during its Grand Finale at the end of mission will provide complementary measurements to place better constraints on the ionosphere as a mass source at Saturn. These studies will also provide a valuable context before Juno's arrival at Jupiter.
